# Functional neurological disorder and multiple sclerosis: a systematic review of misdiagnosis and clinical overlap

**DOI:** 10.1007/s00415-021-10436-6

**Published:** 2021-02-21

**Authors:** Dennis Walzl, Andrew J. Solomon, Jon Stone

**Affiliations:** 1 Centre for Clinical Brain Sciences, University of Edinburgh, Chancellor’s Building, Edinburgh, UK; 2 Larner College of Medicine at the University of Vermont, Burlington, VT USA

**Keywords:** Multiple sclerosis, Functional neurological disorder, Conversion disorder, Psychogenic, Misdiagnosis

## Abstract

Multiple sclerosis (MS) and functional neurological disorder (FND) are both diagnostically challenging conditions which can present with similar symptoms. We systematically reviewed the literature to identify patients with MS who were misdiagnosed with FND, patients with FND who were misdiagnosed with MS, and reports of patients with both conditions. In addition to FND, we included studies of patients with other functional and psychiatric disorders where these caused symptoms leading to investigation for or a diagnosis of MS, which in a different context would likely have been labeled as FND. Our review suggests that MS is one of the most common causes of misdiagnosis of FND and vice versa. We discuss the clinical errors that appear to result in misdiagnoses, such as over-reliance on psychiatric comorbidity when making a diagnosis of FND or over-reliance on neuroimaging for the diagnosis of MS, and practical ways to avoid them. Comorbidity between these two conditions is also likely common, has been poorly studied, and adds complexity to diagnosis and treatment in patients with both MS and FND. Misdiagnosis and comorbidity in a landscape of emerging evidence-based treatments for both MS and FND are issues not only of clinical importance to the care of these patients, but also to treatment trials, especially of MS, where FND could be a hidden confounder.

## Introduction

Multiple sclerosis (MS) and functional neurological disorder (FND) are both diagnostically challenging conditions presenting at times with similarly disabling paroxysmal, fluctuating, and multifocal neurological symptoms. They also share a similar epidemiology, with a preponderance of female to male patients, and both are common conditions in neurological practice [1].

Misdiagnosis of MS or FND causes harm to patients, most generally in the form of a delay to diagnosis and initiation of proper treatment. Misdiagnosis of FND in patients who have MS may result in irreversible disability as a consequence of delays in the initiation of disease modifying therapy (DMT). However, DMTs carry unnecessary side-effects and risks for patients with FND who are misdiagnosed with MS. Patients with FND who are misdiagnosed with MS have a prognosis similar to MS [2] but which may improve with more modern evidence-based treatment [3]. Misdiagnosis can also cause psychological harm in patients if they partially shape their identity around a diagnosis of MS or FND that they are later informed is incorrect [4, 5].

FND may also present as a comorbid condition in patients with an accurate diagnosis of MS, in which case both conditions warrant treatment. Failure to detect comorbidity between FND and MS may result in unnecessary treatment of misdiagnosed relapses, and unwarranted escalation to higher risk DMTs in patients with MS, and could also present an important confounder for MS research efforts that include assessment of clinical disability status.

In several previous studies, we noticed that when people who have MS are initially misdiagnosed, FND is often one of the conditions for which it is mistaken [6]; and when people with FND are misdiagnosed, often the erroneous diagnosis is MS [7]. Our findings prompted us to search the literature for further data concerning the diagnostic overlap between MS and FND.

Our aim in this review is to summarize what is known about how often FND is misdiagnosed in patients with MS, how often MS is misdiagnosed in patients with FND, as well as comorbidity between these two disorders. We discuss potential sources of error in the diagnosis of each condition, and implications for both clinical care and future research.

## Methods

We searched English-language studies on MEDLINE from 1965 to April 2020 using the search:

("diagnostic error*" OR "misdiagnosis") AND ("multiple sclerosis" OR ("functional neurological disorder*" OR "functional disorder*" OR "conversion disorder*" OR "somatization" OR "psychogenic" OR "dissociative" OR "hysteria"))

The abstracts of 512 papers yielded by this search were screened for relevance. We excluded case reports. We hand searched references of articles identified for additional studies relevant to this review. We included any study that focused on a diagnostic change between MS and FND, or their comorbidity. In addition to FND, we also included and reported studies reporting patients misdiagnosed as having another functional disorder (including fibromyalgia), a psychiatric disorder, or “non-specific symptoms”. Patients are usually suspected of having MS because they have motor or sensory symptoms. We looked at a broader range of conditions on the grounds that in many cases, it seems likely that clinicians may have made no diagnosis (e.g., non-specific symptoms) or focused on a comorbid disorder that they considered to be relevant or was perhaps dominant, such as anxiety or fibromyalgia, even though those diagnoses do not encompass the type of motor and sensory symptoms that typically lead to the evaluation for, or the diagnosis of, MS. For example, if a patient received a diagnosis of MS, but later was found not to have MS but in fact depression, it seems likely that in a different context, a diagnosis of FND would have been made, as depression could not have otherwise caused neurological symptoms suggestive of MS.

### Patients with multiple sclerosis who are misdiagnosed with functional neurological disorder

FND, also referred to as psychogenic, dissociative, and conversion disorder, consists of motor, sensory or seizure symptoms that can be identified as relating to a functional disorder. The core diagnostic feature in motor and sensory presentations is the finding of impairment in voluntary motor or sensory function which can be shown under other circumstances to be intact. A positive Hoover’s sign of leg weakness or positive tremor entrainment test are examples [1]. A number of studies, particularly if the numerous terms used to describe FND are considered, have found that MS may be misdiagnosed as FND (Table 1).

**Table 1:** Studies reporting patients with multiple sclerosis who were initially misdiagnosed with functional neurological disorder (FND), functional disorder or psychiatric disorder

Cohort studies of multiple sclerosis					
Study	Year data collected	Methodology	Misdiagnosed total	MS misdiagnoses which were:	
				FND, functional disorder or psychiatric disorder	FND alone
Skegg et al. [13]	1976 and 1981	Review of the psychiatry records of 91 patients with MS in New Zealand	8/91 (9%)^a^	8/8 (100%)	4/8 (50%)
Levin et al. [14]	2003	Survey of 50 patients with MS in Israel regarding misdiagnosis before confirmation of MS	29/50 (58%)	9/29 (31%)	Not stated
Aires et al. [15]	2010–2015	Review of causes of diagnostic delay in 285 patients with MS in Portugal	128/285 (45%)	30/128 (23%)	Not stated
Ivaniuk et al. [16]	2007–2018	Review of records of 128 patients with MS in Ukraine for initial misdiagnosis	53/128 (41%)	7/53 (13%)	Not stated

Cohort studies of functional neurological disorder				
Study	Year of original diagnosis	Methodology	Misdiagnosed total	Patients with MS among patients misdiagnosed as FND
Stone et al. [7]	1949–2001	Systematic review of studies on diagnostic outcome of adults with a diagnosis of FND (or older synonyms)	123/1466 (8%; 4% since 1970)	6/68 (9%)
Stone et al. [17]	2002–2004	Prospective cohort study of patients with medically unexplained symptoms as rated by neurologist with 19-month follow-up	4/1030 (0.4%)	1/4 (25%)
Gelauff et al. [18]	2000–2003	Prospective cohort study of patients with functional limb weakness with 14-year follow-up	1/89 (1%)	1/1 (100%)

^a^This study only looked at diagnostic error in patients who had attended psychiatry clinics

Patients with MS often experience a period of diagnostic delay prior to receiving a diagnosis of MS [8]. The reasons for this are multifactorial, and often involve systemic barriers to healthcare access. Although many studies have reported such diagnostic delay in MS care [8–12], only a few have documented specific misdiagnoses that were responsible for such delays (Table 1).

In a 1988 study, researchers identified all patients with a diagnosis of MS in prevalence assessments in 1976 and 1981 in a region of New Zealand, and found that out of ten who had initially been seen by psychiatrists with symptoms attributable to MS, eight were diagnosed with a variety of psychiatric diagnoses for symptoms such as weakness, balance incoordination, sensory symptoms, and vision loss [13]. Four (50%) were given a diagnosis of “hysterical conversion” or similar. It is possible that these particular misdiagnoses might have been prevented using current MS diagnostic criteria that incorporate imaging techniques. However, similar findings were noted from an MS tertiary care center in Israel in 2003. 29 out of 50 (58%) consecutive MS patients self-reported having initially been given 41 different diagnoses prior to a diagnosis of MS; of those 29, 9 (31%) were diagnosed with a psychiatric disorder including anxiety, somatization, or conversion disorder (FND) [14]. In this study, eight additional patients reported that a physician had diagnosed them with “no problem” despite presenting with motor or sensory symptoms, and perhaps they would have been more explicitly diagnosed with FND by a different provider. In a 2019 study, 30 (23%) of 128 patients with MS at five tertiary care centers in Portugal had received psychiatric diagnoses prior to a diagnosis of MS [15]. Similarly, in a 2020 study at a tertiary care center in Ukraine, 53 patients were initially misdiagnosed before receiving the correct diagnosis of MS, including one (2%) with generalized anxiety disorder and six (11%) with “other neuropsychiatric disorders” [16].

Further information about the misdiagnosis of MS as FND comes from cohort studies of FND (Table 1). A systematic review of diagnostic change in FND, incorporating 27 studies and 1466 patients with a mean duration of 5 years going back to the 1950s, found that misdiagnoses of FND had been stable at approximately 4% since 1970. Revised diagnoses were reported in 68 out of 123 misdiagnosed patients; the most common were epilepsy (19%), multiple sclerosis (9%), and movement disorders (9%) [7]. In a subsequent prospective cohort study of patients referred to neurology outpatient clinics in Scotland, only 4 (0.4%) out of 1030 patients with FND acquired a subsequent unexpected new diagnosis which better explained the original symptoms, one of whom had MS [17]. In a 14-year prospective case–control study of 89 patients with functional limb weakness published in 2019, the only person who had a diagnosis at follow-up which in hindsight may have been a better explanation for their symptoms had been diagnosed with MS. This patient had comorbid FND and MS [18]. While these studies demonstrate low absolute numbers of patients with MS who were initially misdiagnosed as having FND, large cohort studies of FND focused on diagnosis are uncommon. Unlike MS cohort studies, these studies employed a restricted and more accurate definition of FND, whereas in the MS literature, functional disorders and psychiatric disorders are often grouped together with inconsistent terminology.

It is difficult to provide a reliable estimation of the frequency of misdiagnosis of MS as FND owing to heterogeneity in design, outcome measures, diagnostic criteria, and terminology used. As a rough guide, from the FND cohort studies presented here, we would summarize that the overall misdiagnosis rate of FND is probably between 1 and 4% and that around 10% of these misdiagnoses may be MS related; in other words, around 0.1–0.4% of people with FND.

### Patients with functional neurological disorder who are misdiagnosed with multiple sclerosis

#### Patients with functional and psychiatric disorders suspected of having MS

Studies reporting a diagnostic change in patients “suspected” of having MS are not clearly a misdiagnosis per se, since in many cases while MS is being entertained as a diagnosis, evaluation remains ongoing. However, if a sufficient number of patients referred for evaluation of suspected MS are found to have FND, it is reasonable to assume that some proportion of such patients are at risk for misdiagnosis of MS. Multiple studies spanning three decades reporting referral patterns to academic MS centers in several countries have consistently demonstrated that patients with FND are indeed frequently referred for evaluation for a diagnosis of MS (Table 2).

**Table 2:** Studies reporting final diagnoses in patients referred for evaluation to tertiary MS centers

Study	Year data collected	Centre where evaluation of MS took place	Patients with diagnoses other than MS	Proportion of diagnoses other than MS which were FND, functional disorder or psychiatric diagnoses
Murray and Murray [19]	1979–1983	Tertiary center, Canada	52/400 (13%)	“Psychiatric conditions”: 14/52 (27%) [anxiety (*n* = 6), depression (*n* = 4), hysteria (*n* = 2)]
Nielsen et al. [22, 23]	1998–2001	Tertiary center, Netherlands	116/377 (31%)	No diagnoses of FND or psychiatric disorders “No certain diagnosis could be made”: 87/116
Carmosino et al. [20], Brousseau et al. [21]	2001–2003	Tertiary center, United States	186/281 (66%)	“Possible psychiatric”: 63/186 (34%) [somatoform (*n* = 48), mood disorders (*n* = 21), anxiety (*n* = 7)]
Rolak and Fleming [24]	2004–2007	Tertiary center, United States	70/142 (49%)	“Psychiatric”: 53/70 (76%)
Bichuetti et al. [25]	1994–2013	Tertiary center, Brazil	495/1599 (31%)	Not specified “Other diagnosis” 108/495
Yamout et al. [26]	2011–2016	Tertiary centers, Lebanon and Kuwait	131/431 (30%)	“Psychogenic”: 19/131 (15%)
Calabrese et al. [27]	2014	22 tertiary centers, Italy	163/667 (24%)	Total: 9/163 (6%) [“psychiatric” (*n* = 5), “fibromyalgia” (*n* = 4)]
Clarke et al. [28]	2015–2018	Tertiary center, United Kingdom	23/35 (66%)	FND or functional sensory symptoms: 3/23 (13%)

In a study of 400 consecutive patients referred to a tertiary MS research unit between 1979 and 1983 (and thus predating the common use of MRI in MS diagnosis) in Canada, 14 out of 52 (27%) patients who did not have MS had psychiatric conditions, including two with “hysteria” [19]. In a retrospective review of 281 new patient evaluations at a tertiary MS center in the United States between 2001 and 2003, 63 out of 186 (34%) patients who did not have MS or possible MS had “possible psychiatric disease” [20]. These patients with psychiatric diagnoses had one or more of “somatoform disorders” (76%), mood disorders (33%), and/or anxiety disorders (11%) [21]. Conversely, there were no diagnoses of FND or psychiatric disorders in a Dutch retrospective review from 1998 to 2001, where 116 out of 377 (31%) consecutive patients referred for a second opinion to a tertiary MS center had diagnoses other than MS or demyelinating disease [22]. Of these patients, 29 (25%) had another neurological disease, none of whom had FND or psychiatric disorders (although five “single diagnoses” were not specified). This study is particularly limited because in a significant proportion of patients, including 80% at follow-up 7 years later, no diagnosis could be identified [23].

In an American tertiary MS center from 2004 to 2007, the most common diagnosis was “psychiatric disease” in 53 out of 70 (76%) patients who had been referred for MS evaluation but did not have MS [24]. A retrospective review of all patients presenting to a Brazilian demyelinating disease center from 1994 to 2013 found that 495 out of 1599 (31%) did not have MS, of whom 108 (22%) had “other diagnosis”, which included psychiatric disorders, although the proportion was not specified [25]. In Lebanon and Kuwait between 2011 and 2015, the most common alternative diagnosis in 131 patients referred with clinical or radiological suspicion of MS to two tertiary MS centers for diagnostic confirmation of MS, who did not have a confirmed diagnosis of MS, was “psychogenic” in 19 (16%) [26].

A study across 22 MS centers in Italy published in 2019 included 667 patients who were referred for suspected MS and followed up for up to three years if their diagnosis remained uncertain. Of 163 patients who did not have MS or clinically isolated syndrome, nine (6%) had psychiatric or functional diagnoses: five had “psychiatric disorder” and four had fibromyalgia [27]. Notably, this study also highlighted a large number of patients with “nonspecific neurologic symptoms” associated “with atypical MRI” (*n* = 10) and “with atypical MRI lesions of suspected vascular origin” (*n* = 40). These are likely to represent a heterogenous group so we have not included them in Table 2, although at least some may have attracted a diagnosis of a functional disorder in other settings. Lastly, in a recent prospective pilot study evaluating an emerging MRI diagnostic biomarker for MS (“central vein sign”) in patients with diagnostic uncertainty, three out of 23 (13%) patients who did not have MS had FND or functional sensory symptoms [28].

In summary, at tertiary MS centers across a wide geographic distribution and over a long duration of time encompassing numerous revisions to MS diagnostic criteria, patients who ultimately had psychiatric disorder, or functional disorder including FND, have been consistently referred for evaluation for a diagnosis of MS. The frequency of such referrals of patients with FND for MS diagnostic evaluations raises the chance of MS misdiagnoses in these patients.

#### Patients with functional neurological disorder misdiagnosed as having multiple sclerosis

In a 2012 survey of 122 MS specialists, 95% reported that they had evaluated in the last year at least one patient who they strongly felt did not have MS despite having carried a diagnosis of MS for at least a year [29]. Many had seen numerous such patients over the preceding year. When asked to recall alternate diagnoses in such misdiagnosed patients, the fourth most common disorder reported was psychiatric disorder (45%) and the sixth was fibromyalgia (31%).

Several studies of varying size and methodological approaches have described characteristics of patients misdiagnosed with MS, including numerous patients with final correct diagnoses of functional and psychiatric disorders (Table 3).

**Table 3:** Studies reporting patients with functional or psychiatric disorder misdiagnosed as MS

Study	Year data collected	Methodology	Total misdiagnosed	Proportion misdiagnosed with FND, functional disorder or psychiatric disorder
Hankey and Stewart-Wynne [30]	1981	Reviewed records of patients either diagnosed with or told they might have MS	69/387 (17%)	All categories: 35/69 (51%) Functional disorder (*n* = 16), anxiety/hyperventilation (*n* = 14), hysterical conversion (*n* = 3), depression (*n* = 2)
Rudick et al. [31]	1986	Case series of ten patients who were misdiagnosed with MS	10 (n/a)	“Hysteria”: 1/10 (10%)
Poser [32]	1997	Review of patients with MS diagnosis referred for second opinion	130/366 (36%)	All categories: 36/130 (28%) Chronic fatigue syndrome (*n* = 28), post-traumatic syndrome (*n* = 5), psychiatric disorders (*n* = 3)
Walzl et al. [34]	2002–2004	Prospective cohort study of new neurology outpatients including 209 with MS	9/209 (4%)	All categories: 3/9 (33%) Anxiety (*n* = 2), chronic fatigue syndrome (*n* = 1)
Solomon et al. [6]	2014–2015	Multicenter case series comprised of patients misdiagnosed with MS	110 (n/a)	All categories: 28/110 (25%) Fibromyalgia (*n* = 16), conversion or psychogenic disorder (*n* = 12)
De Seabra et al. [36]	2009–2016	Retrospective study reviewing patient records at an MS clinic between 2009 and 2016 by applying 2010 McDonald criteria	44/635 (7%)	All Categories: 2/44 (5%) Dissociative disorder (*n* = 1), fibromyalgia (*n* = 1)
Kaisey et al. [35]	2016–2017	Patients with a prior established diagnosis of MS were reviewed in clinic and evaluated for fulfilment of 2010 McDonald criteria	43/241 (18%)	Fibromyalgia: 2/43 (5%)

In a 1981 study including 69 patients misdiagnosed with MS, 35 (51%) had a functional or psychiatric diagnosis: 16 had a functional disorder, 14 had anxiety/hyperventilation, three had “hysterical conversion”, and two had depression [30]. A 1986 case series describing ten patients who were misdiagnosed with MS found one patient whose final diagnosis was “hysteria” [31]. A 1997 study of 366 patients with a diagnosis of MS referred for a second opinion to Charles Poser found 130 misdiagnosed patients, including 28 (22%) with chronic fatigue syndrome and eight (6%) with “psychiatric disorder” or “post-traumatic syndrome” [32]. Of note, these studies predate the incorporation of MRI into MS diagnostic criteria in 2001 and may be less representative of contemporary misdiagnosis.

In the Scottish Neurological Symptoms Study (SNSS), 9 out of 209 (4%) patients with a diagnosis of MS or demyelination [33] at baseline had an alternative diagnosis at 18 months follow-up, of whom three (33%) had a functional disorder or psychiatric diagnosis at follow-up (anxiety and chronic fatigue syndrome) [34]. In a 2016 multicenter case series reporting 110 patients misdiagnosed with MS, 12 (11%) had “conversion or psychogenic disorder”, the fourth most common diagnosis. Also of note, 16 (15%) were diagnosed with fibromyalgia, and 13 (12%) with non-specific or non-localizing neurologic symptoms with abnormal MRI [6]. In contrast, in a 2019 study in California, there were no cases of FND among 43 misdiagnosed MS patients referred to two MS tertiary care centers, although there were two cases (5%) of fibromyalgia [35]. An additional recent study in 2020 in Portugal reported 44 misdiagnoses in 635 patients (7%) referred to an MS clinic between 2009 and 2016 [36]. Of those, one had dissociative disorder and another fibromyalgia, and nine had non-specific MRI changes with unclear explanations for their symptoms.

Once again, it is hard to distill the data to a ‘headline figure’, but it can be seen that the frequency of MS misdiagnosis for all conditions ranged from 4 to 36%. The differential diagnosis of MS is broad, but within this misdiagnosed group, functional or psychiatric disorders were consistently seen, making up between 5 and 51% of patients with these diagnostic errors. The estimates therefore seem somewhat higher in this direction than for patients erroneously misdiagnosed as FND.

### Sources of clinical error in misdiagnosis

The literature reviewed above supports that the misdiagnosis of FND as MS, and vice versa, is not infrequent. Both FND and MS can result in disabling paroxysmal or fluctuating neurological symptoms in young adults, and accurate diagnosis in both disorders relies on a skilled clinical assessment. There remains no highly specific biomarker for either MS or FND. However, consensus clinical criteria for MS have performed with high accuracy when used correctly [37]. Diagnostic criteria for FND based on DSM-5 emphasize finding evidence of clinical incompatibility with recognized diseases, which in practice requires the presence of positive signs of internal inconsistency and incongruity [1]. Clinical practice suggests that FND diagnoses do generally remain stable [7, 17, 18], but more work needs to be done to define the disorder and its many symptoms and subtypes.

Clinician reliance on a history of psychiatric comorbidity or of adverse experience is one of the most common reasons for an incorrect diagnosis of FND [38]. Such factors may be important in formulation or treatment when present, but are so common that they are not of diagnostic value. Additionally, failing to adequately rely on clear positive clinical examination signs of FND to make the diagnosis [1], or placing weight on a “bizarre” presentation which the clinician finds hard to explain [38] are common errors from our experience. Conversely, patients who are older, male and do not conform to stereotypes about FND may experience over-diagnosis with disease diagnoses such as MS.

Data from studies focused on MS [6, 35] suggest that misapplication and misinterpretation of MS diagnostic criteria may be responsible for many cases of misdiagnosis. The McDonald criteria require successive clinical assessments. In cases of FND misdiagnosed as MS, it is likely that the McDonald criteria are applied in patients presenting without one of the required “typical syndromes” for MS, “objective evidence” on neurological examination or paraclinical testing of a corroborating central nervous system lesion. Instead, an over-reliance on brain MRI imaging abnormalities that can be attributed to a variety of other common causes (including small vessel ischemic disease, migraine, or healthy aging) that fulfill the MRI portion of the McDonald criteria, accompanied by non-specific or non-CNS localizing neurological symptoms, may often fit the profile of patients with FND misdiagnosed with MS. Conversely, the widespread adoption of MRI for the diagnosis of MS that has occurred since the completion of a number of the above studies may have resulted in fewer patients with MS having been misdiagnosed with FND.

Neither MS nor FND should be approached as a diagnosis of exclusion. The clinical assessments required to fulfill the McDonald criteria, and specific examination findings in FND (i.e., positive signs) increase specificity for a diagnosis of each disorder. Diagnosing MS in patients with MRI abnormalities and with no better explanation for their neurological symptoms increases the risk of misdiagnosis. Similarly, making a diagnosis of FND in a patient with a normal MRI and unexplained non-specific neurological symptoms is incorrect and likely to lead to misdiagnosis.

### Comorbidity of functional neurological disorder and multiple sclerosis

Is a diagnosis of FND in a patient with MS always an error? Most neurologists specializing in MS recognize that they commonly see patients who clearly have an accurate diagnosis of MS, yet also have symptoms and/or signs which suggest that they have a comorbid functional disorder. This kind of FND comorbidity is well recognized for other neurological conditions. For example, up to 20% of patients with dissociative non-epileptic seizures have a history of, or comorbid, epilepsy [39], and studies of Parkinson’s disease are increasingly highlighting FND as a comorbidity, especially in the prodromal period [40].

Neurologists as far back as Charcot recognized that patients with MS and functional disorders could be intertwined. Gowers wrote that “hysterical and emotional disturbance are common (in MS), even in men” [41]; Oppenheim wrote that “Multiple sclerosis is frequently associated with hysteria” [42]; and Russell Brain wrote in 1930 that “Hysterical symptoms, such as pareses and ataxia, seem to occur more often in association with disseminated sclerosis than with any other organic disease of the nervous system” [43]. Wilson, on the other hand, viewed “hysteria” and multiple sclerosis as separate, and although he recognized that “hysteria” was common, when diagnosed in the early stages, or “pre-disseminated” form of MS, he thought it represented a misdiagnosis [44].

Although widely acknowledged, there is scant data on the prevalence of FND comorbidity with MS [45, 46]. In a study of 366 patients with MS in Denmark in the 1970s and 80s, five (1%) had psychiatric admissions for “hysteric” neuroses in relation to and after the onset of MS [47]. A case series of four similar patients with both MS and “hysteria” was published in JAMA in 1980 [48]. In a 2011 study of new neurology outpatients with a diagnosis of neurological disease, 32 out of 252 (13%) patients with MS were rated by the neurologist as having symptoms only “somewhat” or “not at all” explained by that disease [33]. One unpublished conference abstract from 2019 reported that 7.5% of 120 German MS patients had “inconsistent findings that could not be explained neurologically” [49].

Studies of MS “pseudorelapses” in children [50] and adults [51, 52] have highlighted that FND can be a potential cause of an apparent MS relapse. Although relapse was not specified as a reason for the visits, “non-organic” presentations accounted for 12 out of 371 (3%) rapid access visits to an MS service in Wales, UK [52].

The comorbidity of MS with psychiatric diagnoses has been more extensively studied. A comprehensive systematic review of 118 studies on psychiatric comorbidities in MS found that psychiatric comorbidities were common; in particular, the prevalence of diagnosed anxiety and depression were estimated at 21.9 and 23.7%, respectively [53]. A study of comorbidity in patients with MS found that the 668 patients who also self-reported mental comorbidities experienced a delay to diagnosis of MS, and were more likely to have severe, rather than mild, disability two years after diagnosis [54]. A nationwide cohort study in Denmark did not find a statistically significant increase in diagnostic delay in patients with psychiatric comorbidity, perhaps due to a small number of patients (*n* = 75), but there was a significantly increased mortality [55]. It is important to understand that FND and functional disorders are not synonyms for psychiatric disorder; they are conditions in their own right that do occur without psychiatric comorbidity. Nonetheless, FND and functional disorders also are known for a high frequency of psychiatric comorbidity as well as disturbances in emotional regulation that have also been found in MS [56]. Therefore, the presence of depression or anxiety could add to both the misdiagnosis of FND and MS.

In conclusion, the topic of FND and MS comorbidity is poorly researched, but the studies we have do suggest it occurs, and following a renaissance in FND diagnosis and evidence-based treatment, is now worthy of more careful study.

### Clinical care and research

What are the consequences of misdiagnosing FND in a patient with MS? Difficulties will be especially magnified if the neurologist making the original FND diagnosis did so in a way that failed to validate the patient’s symptoms or provide onward treatment. Whereas in the past, missing a diagnosis of MS could have been said not to have led to a change in prognosis, that is no longer the case with newer disease modifying agents [57].

The implications of misdiagnosis of MS in patients who have FNDs include unnecessary treatment of MS and its accompanying side-effects and risks, delay to diagnosis and treatment of FND, and psychological harm as a result of misdiagnosis. In one study, 31% of patients misdiagnosed with MS experienced unnecessary morbidity as a result of misdiagnosis, most commonly due to unnecessary immunomodulatory therapy and treatment side-effects [6]. Traditionally, neurologists may have considered that accidentally diagnosing MS in someone with FND was less harmful because of a lack of established treatment options for FND. However, the scientific landscape around FND has changed considerably in the last 10 years [1]. Patients with FND erroneously diagnosed with MS miss out on increasingly evidence-based treatment for their functional symptoms. For example, a randomized controlled trial of specialized physiotherapy for functional motor symptoms found that 72% of patient had improvements in mobility sustained at 6 month follow-up compared to 18% in a control arm having non-specialized physiotherapy [3].

The diagnosis of a neurodegenerative disease such as MS can have a life-altering psychological effect on patients. Patients can shape their identity around their diagnosis, and it is difficult for neurologists to remove a diagnosis of MS without concern of causing yet more harm to the patient [4, 5]. This is perhaps especially the case when the new diagnosis is one of a functional disorder, when the neurologist may feel less equipped to help than they were with an MS diagnosis [58].

Identifying comorbid FND and MS can lead to fundamental differences in treatment. The treatment of FND depends firstly on establishing with the patient that they have a condition which is potentially reversible, and related to a problem in the “software” of the nervous system, rather than a “hardware” problem as seen in established MS. Second, the type of physical rehabilitation that has been developed for FND uses a transparent explanatory model for the disorder which takes advantage of the inconsistencies seen on examination and turns them into therapeutic opportunities during therapy [3]. So, a patient with FND may be discouraged from thinking too hard about their walking and to actively use distraction techniques to improve function, whereas in therapy for someone with MS, the patient may be asked to concentrate hard on the affected limb. Furthermore, recognition of FND comorbidity in patients with MS has important consequences for the assessment of MS relapses. In such patients, additional paraclinical testing, such as MRI, might be necessary to confirm “objective evidence” of a CNS lesion responsible for symptoms or challenging neurological exam findings.

Lastly, the identification of patients with MS and FND comorbidity may have a further benefit for MS research. Patients with FND comorbidity may increase “signal to noise ratio” in clinical trials of new therapies for MS with subjectively rated clinical outcome measures. If reliable ways could be found to exclude such patients, this could lead to better data from trials of MS therapeutic agents, a concept that has also been proposed in relation to functional cognitive disorders and dementia trials [59].

## Conclusions

MS and FND are common conditions that are not infrequently mistaken for one another, and which may co-exist more often than is currently acknowledged. Improved clinical recognition and broader research efforts focused on the overlap of these two disabling disorders has potentially important consequences for the care of patients with MS and FND and for treatment studies in both conditions.

## Data Availability

Not applicable.
